# The Emotional Experience of Caring for Children in Pediatric Palliative Care: A Qualitative Study among a Home-Based Interdisciplinary Care Team

**DOI:** 10.3390/children10040700

**Published:** 2023-04-09

**Authors:** Patricia Rico-Mena, Javier Güeita-Rodríguez, Ricardo Martino-Alba, Marina Castel-Sánchez, Domingo Palacios-Ceña

**Affiliations:** 1 Physical Therapy and Health Sciences Research Group, Faculty of Sport Sciences, Department of Physiotherapy, Chiropody and Dance, Universidad Europea de Madrid, 28670 Madrid, Spain; 2 International Doctorate School, Rey Juan Carlos University, 28008 Madrid, Spain; 3 Department of Physical Therapy, Occupational Therapy, Physical Medicine and Rehabilitation, Research Group of Humanities and Qualitative Research in Health Science, Rey Juan Carlos University, 28922 Alcorcón, Spain; 4 Pediatric Palliative Care Unit, Hospital Universitario Infantil Niño Jesús, 28009 Madrid, Spain

**Keywords:** pediatrics, palliative care, personal satisfaction, compassion fatigue, burnout, professional, attitude of health personnel, emotions, child, qualitative research

## Abstract

The healthcare providers caring for children with life-threatening illnesses experience considerable compassion fatigue. The purpose of this study was to describe the feelings and emotions of professionals working in an interdisciplinary pediatric palliative home care team. A qualitative case study was conducted, comprising 18 participants. A purposeful sampling technique approach was used including the home-based interdisciplinary pediatric palliative team. Data were collected via semi-structured interviews and researchers’ field notes. A thematic analysis was performed. Two themes emerged: (a) changing life for the better, which described how professionals value life more and helping children and families provides compassion satisfaction, which is comforting and explains their dedication to care; (b) adverse effects of work highlighted the emotional burden of caring for children with life-limiting or life-threatening illnesses, which can affect their job satisfaction and may lead to burnout, showing how experiencing in-hospital child deaths with suffering leads professionals to develop an interest in specializing in pediatric palliative care. Our study provides information on possible causes of emotional distress in professionals caring for children with life-threatening illnesses and highlights strategies that can help them to reduce their distress.

## 1. Introduction

Pediatric palliative care (PPC) addresses the needs of children and adolescents with life-limiting/life-threatening conditions or terminal disease and includes family care [1,2]. Professionals among the interdisciplinary PPC team (ID-PPC) are responsible for ensuring the quality of life of the child and family and preventing and alleviating suffering by determining the goals of care, managing symptoms, and guiding decision-making in complex or critical situations involving end-of-life care and bereavement after the child’s death [2]. ID-PPC professionals are continuously exposed to emotionally demanding clinical experiences [3,4]. These experiences are prolonged due to longer care periods in PPC compared to adult palliative care [5], creating closer relationships with patients and their families that increase the practitioner’s emotional burden [4]. In addition, as a health professional, facing the death of a child is more distressing and traumatic than experiencing the death of an adult [4,6,7].

Compassion fatigue, burnout, and compassion satisfaction are three phenomena that can disrupt both the personal well-being and job performance of healthcare professionals [3]. Compassion fatigue is the emotional distress experienced by professionals due to continuous contact with the suffering of patients and arises from the feeling of empathy towards the person who suffers [8]. Burnout is a syndrome characterized by emotional exhaustion, depersonalization towards patients, and a reduced sense of personal fulfillment, and it manifests itself in professional dissatisfaction [9]. Compassion satisfaction is the emotional satisfaction derived from caring for others [10].

The few studies available on the prevalence of compassion fatigue, burnout, and compassion satisfaction among in-patient PPC professionals show low levels of compassion fatigue and burnout, and high job satisfaction [3,11,12,13]. The job satisfaction of professionals appears to be a key factor in the effectiveness and quality of palliative care [3,12], and, therefore, a deeper understanding of the experiences and impact of this care work is required. Previous studies have focused mainly on identifying predictors of compassion fatigue, burnout, and compassion satisfaction in PPC professionals through the use of specific questionnaires [3,12] or by exploring their overall experiences in providing PPC at the end of life through qualitative studies [14]. However, to date, there are no previous qualitative studies describing the emotions and feelings of professionals who integrate home-based ID-PPC throughout the care process during the entire trajectory of the child’s illness and not only at the end of life. Their identification, and the coping strategies used in PPC to manage the emotional burden of caring for a child with life-limiting/life-threatening conditions may help to implement preventive measures or approaches that improve the well-being of professionals, and, thus, the care they provide to their patients [14]. The purpose of this qualitative study was to describe the feelings and emotions of professionals working in a home-based ID-PPC who carry out their healthcare activity within the public health system of the region of Madrid (Spain).

## 2. Materials and Methods

This study was conducted according to the Consolidated Criteria for Reporting Qualitative Research [15] and the Standards for Reporting Qualitative Research [16].

### 2.1. Design

A qualitative case study was developed [17,18]. This design enables the investigation of complex phenomena within their real context and to collect information from multiple sources (different professionals) [19]. This design comprises different participants, contexts, places, and moments connected by the phenomenon under study [17,20]. In this study, the phenomenon was the process of care for children included in the Madrid PPC program, as experienced by the different professionals that work in home-based ID-PPC. The main characteristic of qualitative case studies is that they provide an intense analysis of a unit, which is the particular case to be studied, whether it be individual, a group or organization, or, in this case, the ID-PPC of Madrid [19,21]. Currently, PPC in Madrid is provided through the only specialized PPC Unit that exists in this community, which is currently located at the Niño Jesus Hospital.

### 2.2. Study Setting

The specialized PPC Unit of the Niño Jesús Hospital receives approximately 220 new pediatric patients per year. Its activity mainly takes place at the children’s homes, providing palliative care to approximately 90 children at their homes per year, attending approximately 180 episodes of home hospitalization [22]. Two thousand home visits are made annually, with 24-h care provided, and at least 8% of the visits are made in continuous care [22]. Approximately 70% of the children seen suffer from neurological diseases and 30% have oncological diseases [22].

### 2.3. Participants, Sampling Strategies, and Sample

Purposive sampling and snowball techniques were conducted, aimed at including those participants who possessed information that was relevant to the study [23]. The study sample included all professionals who were part of the home-based ID-PPC. The inclusion of all participants is recommended in qualitative studies where it is possible to have access to all participants with relevant information about the case study [24,25].

This study included professionals working within home-based ID-PPC. The inclusion criteria consisted of being a member of the ID-PPC providing home care at the time of the study. The professionals who conformed with the home-based ID-PPC were specialist palliative care doctors, specialist palliative care nurses, specialist palliative care psychologists, specialist palliative care social workers, specialist palliative care physiotherapists, and specialist palliative care administrative staff. All participants were requested to sign the informed consent form.

### 2.4. Data Collection

In-depth interviews and researchers’ field notes were used (Table 1) [24]. Interviews are a data collection tool that enable in-depth information to be obtained from the participants’ perspectives [25]. Interviews allow flexibility which enables researchers to investigate those aspects considered relevant by the participants [25].

A guide of semi-structured questions was used during the interviews, based on the literature [14] and expert knowledge (Table 2). The interviews were conducted in Spanish by a single researcher (PRM) and a physiotherapist with experience in PPC and qualitative research, and were audio-recorded after obtaining permission from the participants.

### 2.5. Data Analysis

A transcription of each interview and researcher notes was made. An inductive thematic analysis of the transcribed texts was conducted by three researchers (PRM, JGR, DPC). The coding and identification of themes was carried out through the reading of the overall text, as well as paragraph by paragraph, and using an in-depth line-by-line analysis. The coding was carried out in distinct phases: (1) Initial codes were generated. (2) Codes were then grouped into categories [25,26]. The categories were then grouped, and sub-themes and themes were identified. (3) Themes were then reviewed, with re-coding and identification of new themes and construction of conceptual maps of the themes. (4) Identification and definition of themes and sub-themes was carried out, establishing their definition [27]. The Atlas-ti^®^ (v.8) qualitative analysis software [28] and Microsoft Excel [29] were used to support data processing and analysis procedures.

### 2.6. Rigor and Quality Criteria

The criteria for guaranteeing trustworthiness produced by Guba and Lincoln were applied [30]. The techniques used to control trustworthiness are reported in Table 3. The use of these methods to increase rigor are compatible with case-study designs [31,32].

### 2.7. Ethical Considerations

This study was approved by the Research Ethics Committee of the Rey Juan Carlos University (code: 2606201710917) and the Clinical Research Ethics Committee of the Niño Jesús Hospital (code: R-0019/17). In addition, this study was conducted in accordance with the principles of the Declaration of Helsinki. Informed consent and permission to record the interviews were obtained from all participants.

## 3. Results

Eighteen participants (12 women, 6 men) took part in this study. The mean age of the home-based ID-PPC members was 38.2 years (SD ± 7.9). Of these, 38.9% were pediatricians, 27.8% were nurses, and the remainder were other health professionals (16.7%, psychologists and physiotherapists), non-health professionals (11.1%, social workers), and there was one non-health member of staff (5.6%, administrative services staff). The mean number of years the professionals had worked at the home-based ID-PPC was 6.0 ± 3.4 years. Regarding the level of specific palliative care training, 50% of the professionals had a Master’s degree, 27.8% had postgraduate courses, 11.1% had training during their rotation as medical residents, and 5.6% were doctors (PhD) in PC.

**Figure 1:**
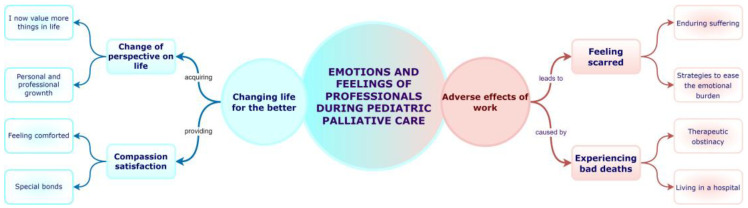
Conceptual map of the study findings.

Two main themes were identified, with four sub-themes and eight categories (Figure 1). In Table S1, examples of participants’ narratives are shown to justify the results obtained, thereby enabling their traceability and credibility [33].

Our results illustrated the emotions experienced by professionals during the provision of pediatric palliative care, including end-of-life care, and how caring for these children can affect job satisfaction and burnout. Knowing the emotional impact derived from palliative care work helps to better understand the concept of PPC and how it is delivered by professionals.

### 3.1. Theme 1: Changing Life for the Better

The home-based ID-PPC professionals reflected on how their dedication to PPC offers them positive benefits such as the ability to make more sense of life, feelings of personal growth, satisfaction, breadth of knowledge, and a more compassionate approach to clinical care.

#### 3.1.1. Sub-Theme: Change of Perspective on Life

Caring for children and families in the palliative context encourages professionals to acquire a different way of seeing and understanding life in a more positive way. They stated that they had experienced changes in their priorities and life goals that contributed to their personal growth. In addition, they described how their PPC experience had helped them to develop professionally.

##### I Now Value More Things in Life

“Valuing life more” was a category highlighted by the professionals. They recognized how their work in PPC had led them to appreciate details of their lives that previously went unnoticed because of their daily occurrence, such as having a family or feeling well. They stated how “working in palliative care has changed their lives” and felt fortunate and grateful that they and their families were in good health.

EPAD1: “I believe that it makes you value other things in your life more, it makes you say: “It’s good that I have a family, it’s good that they are well and it’s good that they are not affected by this type of disease. Because it is a physical and emotional wear and tear, not only for the patient, but also for everything it entails, which influences your professional, family, and social life.”

They described greater resources to “relativize” problems by focusing on the search for solutions.

EPE5: “When you work here, when you see real dramas, you relativize. You relativize because you think that maybe a problem that you may have personally or a problem that may arise at work is not so important; and your priorities change. Your way of understanding the world changes, because you put on other lenses for your life. It teaches you to enjoy small moments that you didn’t notice before.”

Repeatedly facing complex situations of health, disability, or death, and observing the strength of children and families in the face of suffering and illness led professionals to express feelings of admiration, inspiration, learning, gratitude, and privilege.

EPE1: “One of the things I like the most about palliative work is that in the end it brings you face to face with what is most human. I have seen love scenes that I haven’t seen anywhere else. Parents with their children, between siblings, and between couples […] I have some scenes engraved in my head, of parents saying goodbye to their children, talking to them at the moment of death…of a love, of a generosity…that I say: God, how are they capable of doing that?”

##### Personal and Professional Growth

All the professionals experienced a feeling of personal growth as a result of caring for the children and their families. They described how they felt more mature, sensitive to suffering, and tolerant of other people’s attitudes or situations since working in PPC. According to their perception, PPC provided them with the opportunity to work on and improve personal skills such as self-reflection, thinking, communication, and emotional management.

EPE5: “You can stop and think. Having a profession where one of the things you have to do as a professional is to stop and talk to yourself and stop and analyze how you feel and work through your feelings, well, I can’t think of many professions where that’s a requirement.”

Aspects related to the feeling of happiness or a state of mind that contributed to personal growth were identified. The professionals felt more optimistic, kind, and happy as they were able to appreciate and enjoy life more because of the awareness of the immediacy of death.

EPM3: “It changes your outlook on life, if you see the people who work in palliative care, almost all of us tend to be quite nice, kind, and happy people, because I think you learn to value things: those that are important, those that aren’t, and to give them more value. You learn not to leave things for tomorrow; you see people dying every day, so you think: I could be next. You perceive life more intensely.”

Professional enrichment was another finding that contributed to the feeling of personal growth. The professionals reported how the complexity and demands of working in PPC had led them to become better professionals, increasing their motivation for training.

EPE4: “It has changed me on a professional level, because it has made me learn more, it has made me try to be a better nurse, because they are very complex children and they require care that I, as a nurse, have had to learn little by little. Here I have been able to feel like a real nurse working. In other places where I have worked I felt more like a mere dispenser of medication, and here I feel that I can develop my profession much more because parents and children need care and a lot of health education.”

#### 3.1.2. Sub-Theme: Compassion Satisfaction

Among the testimonies of all the professionals, the feeling of compassion satisfaction was related to the benefits they appreciated when applying care.

##### Feeling Comforted

The professionals expressed how comforting it is for them to see how the efforts they make in their work help the children and their families at a fragile and complicated time in their lives that causes them much suffering.

EPM1: “As painful as it is to have a child die, which I think is the most painful thing that can happen to someone, the fact that this makes it a little easier, a little more humane and less painful, comforts me.”

The professionals described the high level of satisfaction they derive from their work with the expression “these children give me life”.

EPE3: “When you give, you are much happier. So, these children have given me life, they die, but they give me life. I am sure that these children leave a legacy here and they leave a purpose, they are here for a reason”.

The professionals expressed that one of the most satisfying aspects of their work was feeling that PPC improves the care of a group of children who, in their opinion, are “largely forgotten” by the health system and do not receive sufficient care outside PPC.

EPE4: “For me, being able to help these families who are the lame limb of the health system, the most forgotten, seems incredible to me; it is one of the things that this service offers me the most…”.

They described the feeling of having contributed something valuable through PPC, making it possible for the families to be accompanied and providing them with a focal resource of professionals who will assume the care of their children without requiring numerous medical specialties.

EPE3: “When our service was created, the families of children with neurological, rare or neurovegetative diseases saw us as a blessing because they were no longer alone in their chronic life process. These children did not have a specialist or a medical care figure or a team to rely on, both psychologically and socially.”

The professionals also highlighted experiences related to the moment of death of the children that they described as “wonderful”. Being able to contribute towards the “good death” of the children, calmly and with their family, and the gratitude shown by the families for this, made the professionals feel satisfied and proud of their work, which compensated for the emotional burden derived from care.

EPM4: “When a family is very afraid of the death of their child, and you think it’s going to be a catastrophe, and then they tell you: he died peacefully, I’m happy despite what happened. Those are things you learn from, it’s a wonderful experience”.

##### Special Bonds

Another finding in relation to the professionals’ satisfaction was the relationships established with the children and families they had served, which they described as “special bonds”.

EPE5: “Besides, it is very enjoyable and creates a very special bond with the families, I find it very enriching, a privilege.”

The implementation of home care and long periods of care contributed to the development of stronger and more durable bonds with children and their families, which motivated professionals to continue their work in the PPC setting.

EPE3: “The relationship is much closer, much more trusting because we enter their homes. The home is the most intimate place in a family, and you go in there, they open their hearts to strangers and ultimately, you establish such a strong relationship that when you leave, they say: I miss you, you are part of my family.”

### 3.2. Theme 2: Adverse Effects of Work

Participants identified how the challenges posed by PPC work can cause them emotional overload or discomfort. In addition, they described negative experiences of death for children and their families, outside of the palliative context, that have had a profound impact on them and caused them suffering.

#### 3.2.1. Sub-Theme: Feeling Scarred

The participants expressed how the complex care of seriously ill children and the care of their families left an emotional mark on them. The integration of support strategies for professionals within ID-PPC seemed to make the difficult task of accompanying the child and family through the process of illness, death, and bereavement more bearable.

##### Enduring Suffering

The professionals used expressions such as “it leaves a scar”, “they are small wounds that you get”, “it gets added to your backpack”, and “it makes a dent” to refer to the cumulative impact of continuous contact with suffering and the end of life and death of the children they care for in the context of palliative care.

EPE3: “There have been times when I needed to take a break and then come back, because it’s really hard. We are dedicated to seeing children and people who suffer, and with these families you have a personal history between you and them, which no one else has, and that is added to your backpack. Therefore, the moment of a child’s death is very sad, however, it is also a very special moment, of introspection, of trying to give peace to that uneasiness that they have, the problem is that it also tires you, more than tiring, it leaves a scar...”

Professionals described the most salient experiences that generated distress and suffering, or had an emotional impact. These included perceiving the suffering of children prior to entering palliative care due to the application of interventions or treatments that maintain a curative approach and do not benefit them; once they entered the PPC program, having difficulties in the control of symptoms that caused suffering due to the complexity of the case; and experiencing the moment of death, witnessing the dead child.

EPM5: “…the first patient that I saw die, I have the face, the expression engraved in my mind. It was in a nursing home, a child with a neurological disease and the nurse who had been on the team for a little longer, you know, she had to pull me along, encouraging me.”

The professionals affirmed that working in PPC is stressful and overloading, and wears out the professional due to repeated exposure to pain and the close relationship with the suffering child and family, whose situation is always going to get worse.

EPE3: “Sometimes I think about it and I say: well, I think I’ve done my job, I’ve been doing it for a long time, let others come and follow... but then I have a conscience that tells me: don’t leave these children, are you going to leave them, are you going to abandon me too? Then you say: I’m not leaving! But many times it’s hard for me because of the work with the families who cry a lot at the end and you say: My God, I can’t do it anymore, I’m going to leave”.

##### Strategies to Ease the Emotional Burden

Peer support among ID-PPC colleagues was another significant finding that could help professionals cope with the emotional burden of “complicated” and “shocking” care work.

EPM6: “It is a different job because it’s a patient that you are going to see continuously, and when he’s ill, which is perhaps when you feel the most burdened, you have to see him even more. Therefore, there is less time to disconnect. So, it’s a more demanding job because it’s a child that you see many times, it’s a family with whom you talk many times about how the disease is worse, the symptoms are less easily controlled. These are very dense issues and you have an overload, but being able to count on another colleague who can stand in for you on the day when your neuron is frayed, in that sense it does help a lot.”

Strategies that favor open communication among professionals are integrated into the ID-PPC’s operating dynamics, encouraging “pain to come to light”, such as weekly team meetings or bereavement and grief sessions.

EPM4: “We also have strategies to get people’s pain out in the open: we have bereavement meetings to be able to express dissatisfactions, doubts, pains related to death, or the relationship with another partner, the family. You have to have strategies for airing unresolved conflicts or grief.”

A sense of humor, companionship, life outside the work environment (family, hobbies, and friends), and periods of rest or disconnection appeared as useful support strategies that provide professionals some emotional support.

EPTS2: “When I became pregnant, I informed the chief of service and told him: I don’t know if I will be able to continue doing this job when I become a mother. Because I thought I was going to put myself in the position of a mother instead of a professional, I was going to see my children dying and I was going to feel very sensitive. Then, in the end, I became a mother and it was very good for me to ask for a leave of absence, because I think this job is a big sacrifice and to have time to rest and to have long vacations, which are necessary in order to truly disconnect, a week’s vacation is not enough.”

#### 3.2.2. Sub-Theme: Experiencing Bad Deaths

Most of the ID-PPC professionals providing home care acknowledged that, prior to working in PPC, they had faced negative clinical experiences in other services surrounding the death of children. Specifically, these bad experiences led them to become interested in PPC, in the hope of finding a form of care that would improve end-of-life care for children and their families. The participants’ narratives illustrated what it means to suffer a “bad death” of a child from the perspective of the professionals: (1) dying alone in the hospital without physical contact and without the company of their parents; (2) lack of information and communication with parents about the imminence of death; (3) poor control of symptoms by applying measures that increased suffering without providing a clear benefit; and (4) “living in a hospital”, depriving them of their family life, and even with some children who are “born and die” at a hospital, without having ever been able to go home.

EPM4: “Here, in intensive care, I experienced very bad deaths with lack of information to the families, deaths without physical contact with the child, and in intensive care in Switzerland I experienced something very different: information, withdrawal of measures, I discovered the accompaniment of death thanks to the professionals there. That made me question things and I said: I want to do something about it when I come back. As I say: here I learned to intubate and there I learned to extubate.”

##### Therapeutic Obstinacy

The professionals stated that they experienced suffering when they witnessed clinical practices that, in their perception, fell into the category of therapeutic obstinacy.

EPE4: “In the ICU I have also seen a lot of obstinance, seeing intubated children in which most of the nursing staff suffered because they were undergoing another transplant, because they were dying badly, and they did not get to palliative care, not even close. What struck me most was the oncology part of the ICU, of transplants, of hemofilters, of going on and on, until they die badly, the parents in a bad way and the children even worse.”

The testimonies coincided with periods of work by professionals in oncology services and pediatric intensive care units. They reflected how in medical specialties with a curative approach it was more difficult for them to accept the irreversibility of a disease process and to stop applying curative treatments.

EPM6: “In oncology patients there is brutal therapeutic obstinacy because it is very difficult to give up. Neurological children are different because they are children who are already born with a serious problem and who have had a serious problem all their lives. A child with cancer is a healthy child and suddenly a devastating disease appears that destroys all the grand schemes created up to that moment, but you do have the memory of a healthy child.”

##### Living in a Hospital

The professionals emphasized how children’s lives prior to being treated by the home care ID-PPC are often marked by emergency room visits, long hospital admissions, and numerous medical appointments that further increase their stay in the hospital, limiting their family life. Experiencing these situations led the professionals to describe deep feelings of sadness, pain, and suffering, and motivated them to train in PPC. The professionals understood PPC as a form of care that looks after the child’s best interests and prioritizes their welfare without forgetting their family.

EPM7: “Seeing the suffering of children in the ICU, meeting many children who were born in a hospital and died in a hospital without going home, and who may have had minimal moments of wakefulness because they had to be sedated for multiple heart surgeries, complications of prematurity, children who have not been able to meet their siblings because they could not leave home and a child cannot enter an ICU, such as a sibling. When I saw that this existed, I said to myself: palliative care should help the child to live as a child! If you know he isn’t going to be cured, don’t condemn him to live in a hospital.”

## 4. Discussion

Our results showed how professionals dedicated to providing PPC experience positive feelings and emotions during care, highlighting personal growth and compassion satisfaction. However, despite describing their work as comforting, they acknowledged a great impact on an emotional level which sometimes takes a toll on them and is exacerbated by experiencing bad deaths.

### 4.1. Theme 1: Changing Life for the Better

Our participants experienced positive changes in life priorities and goals, greater appreciation for life, personal strength, or improvements in personal resources and relationships, in line with the key components of personal growth defined by Tedeschi and Calhoun in five domains [34]. Beaune et al. showed how doctors, social workers, and nurses experienced personal growth, appreciating the good in their lives and being more benevolent when caring for children with life-limiting conditions despite not belonging to an ID-PPC [35]. Benevolence refers to an altruistic and compassionate attitude related to the desire to improve patient care that has been recognized as a beneficial effect of care [35,36], and in our participants it seemed to contribute to their perception of personal growth. The nurses in Conte’s study reported how they had learned to appreciate their lives and the time they spend with loved ones more because of their work in pediatric oncology [37].

In addition, professionals described how working in PPC helped them to be a “better professional”. The bone marrow transplant unit nurses who participated in the Morrison and Morris study identified learning new technical skills, acquiring disease-specific knowledge, and the enrichment provided by daily patient-centered rounds with an interdisciplinary team as opportunities. Like our participants, they felt privileged to care for children in what they considered the hardest time of their lives [38].

In line with our results, previous studies also reported high levels of job satisfaction among PPC professionals [3,11,12,13]. Satisfaction and compassion fatigue are less explored phenomena in healthcare professionals [3,39,40]. Burnout, on the other hand, has a large body of evidence [41,42,43,44,45,46]. However, both compassion satisfaction (25%) and compassion fatigue (18%) were more prevalent than burnout (12%) among the 150 PPC providers who participated in a survey in the United States in 2019. It is essential to promote compassion satisfaction and not just prevent or reduce compassion fatigue or professional burnout [3]. The predictive factors for lower compassion satisfaction scores identified by Kase et al. were physical exhaustion, a personal history of trauma, re-occurring involvement in a clinical situation in which life-prolonging measures were not introduced, and failure to discuss burdensome issues. In our results, personal experiences of suffering or illness in the private life of ID-PPC professionals increased their empathy and detracted from physical exhaustion, appearing as factors that reinforce compassion satisfaction, together with open communication, mutual support within the team, and close relationships with families [14,35,47]. Klassen et al. concluded that one of the main rewards obtained among pediatric oncology professionals was the close relationships they established with parents and the ability to help families throughout the oncology process, including PPC [47]. Conte reported how close relationships fostered a sense of connection with patients in pediatric oncology nurses that helped them make sense of their work, yet intensified their grief at the loss of a child [37].

In addition, the professionals described how experiencing the application of life-prolonging measures without prioritizing the patient’s well-being caused them suffering and moral and emotional discomfort. Therefore, our results suggested that the participation of the ID-PPC in clinical situations where burdensome life-prolonging measures are not introduced was related to a greater satisfaction with the professionals’ compassion, and not with lower rates as reported by Kase et al. [3].

Our findings provided information in this little-studied field of possible causes that explain the high level of job satisfaction in PPC professionals: (1) the humanization of care in the process of illness and death of the child; (2) a feeling of privilege and satisfaction in being able to help parents in the difficult process of living through the illness and death of their children; and (3) that PPC makes it possible for the child to live at home and not in a hospital. Feeling satisfied with their work seemed to help ID-PPC professionals providing home care to manage the emotional burden of caring for children with life-limiting/life-threatening conditions [3,35].

### 4.2. Theme 2: Adverse Effects of Work

The participants acknowledged that it is a stressful and psychologically overloading job that leaves them with “emotional scars”, which was in line with previous studies [4]. Kase et al. reported three predictors of compassion fatigue in PPC professionals: distress about the clinical situation, physical exhaustion, and personal loss [3]. Physical exhaustion also emerged in our study as being associated with the dynamism of home care and 24-h care. Moreover, personal loss was not identified in our results, but similar personal situations related to the feeling of loss were identified, for example, with the professional’s decision to have a child and to empathize with the families in their loss, something that they consider may weaken them and prevent them from doing their work.

In addition, negative emotions and feelings emerged, such as grief, anguish, sadness, and suffering, that were derived from care, but not only in the palliative context. The professionals alluded that the hardest experiences of “bad deaths” were experienced outside palliative care, mainly in oncology or pediatric intensive care units. The concept of a “good death” is of great importance in PPC [48]. Compared to adults, there is less information on what a “good death” means for a child, and the available studies focused on children with cancer [48,49]. Our study provides relevant information, drawing on the experience of the most specialized professionals in pediatric end-of-life care, which contributes to fill this knowledge gap, shedding light on factors and characteristics that may contribute to a negative death for children and their families.

Related to the feeling of distress and discomfort expressed by the professionals in our study, Lee and Dupree also described sadness and grief, rather than moral distress, as the main psychological responses of intensive care personnel when caring for a dying child [50]. Moral distress occurs when you know what the morally right thing to do is, but institutional constraints prevent you from doing it [51]. Nevertheless, a study by Dryden-Palmer et al. revealed that moral distress is a common feeling in pediatric and neonatal ICUs associated with increased depersonalization of care and uncertainty related to end-of-life decision making in a child’s life [52]. In addition, staff working in pediatric intensive care units do not have sufficient emotional support to cope with the grief of children’s deaths [50].

Our results showed how the experience of “bad deaths” in other fields, such as oncology or pediatric intensive care, seems to lead professionals to become interested in the field of PPC and abandon intensive care, as was the case with three of the seven physicians who participated in the study and four of the five nurses.

Similar to our participants, the nurses who participated in the qualitative study by Maytum et al., who were dedicated to caring for chronically ill children, described strategies that helped them to minimize episodes of compassion fatigue and that were useful for preventing burnout: choosing a work environment in accordance with their personal philosophy, changing jobs when necessary, or taking advanced training courses [53]. Jonas and Bogetz revealed how the support provided by ID-PPC to other pediatric services working with critically ill children is critical to prevent professional distress and burnout [54]. Weintraub et al. identified the use of PPC as a determinant of higher compassion satisfaction among U.S. neonatologists [40].

Feeling sadness and grief for the death of the child is assumed by professionals as a sign of humanity and connection with the child and the family [50]. Our results described some strategies integrated into the home-based ID-PPC performance in line with previous recommendations to prevent fatigue and burnout: peer support, open communication, family life [55], and team meetings after complex clinical situations [3]. A sense of humor, positive thinking and attitude, and time away from work can also help professionals unwind [53]. These factors help to understand the higher satisfaction reported by PPC professionals [56,57].

This study provides an important perspective from our geographic region that presents a need for further development of PPC as expressed in the European Atlas of Palliative Care [58]. To our knowledge, this is the first study that specifically examined the feelings and emotions of professionals belonging to a specialized ID-PPC in Spain. In addition, it makes important contributions to the existing literature, mostly focused on pediatric oncology [37,38,47,49] or terminal care [14,50,59]. Most of these studies focused on the experiences of physicians, nurses, and social workers in PPC [35,37,38,47,59], whereas our research included the perspective of the ID-PPC and included psychologists, physiotherapists, and administrative staff.

A limitation of our findings is that they cannot be extrapolated to all ID-PPC professionals involved in home care, due to the nature of the research question and the qualitative design selected.

As future lines of research, we suggest studies to establish the prevalence of compassion fatigue, burnout, and compassion satisfaction among PPC providers in Spain and an in-depth study of the factors associated with the perception of a “good death” from the perspective of other medical specialties involved in the care process, and of parents and children in PPC. Furthermore, studies into how motherhood/paternity can influence professionals caring for children in PPC would be welcome.

## 5. Conclusions

In-depth knowledge of the emotional affect of home-based ID-PPC professionals contributes to understanding the meaning and magnitude of PPC. Incorporating the perspectives of ID-PPC professionals can guide the implementation of measures that counteract professionals’ stress and burnout, and promote their well-being, improving the quality of PPC.

## Figures and Tables

**Table 1:** Data collection instruments.

Participants	Number of Participants	Data Collection Tool	Time (min)
Professionals on the specialized home-based PPC team	7 PCD, 5 PCN, 2 PCP, 2 PCSW, 1 PCPT, 1 PCA.	18 semi-structured interviews and field notes	Total: 940 min; mean: 58 min

PCD: Specialist Palliative Care Doctor; PCN: Specialist Palliative Care Nurse; PCP: Specialist Palliative Care Psychologist; PCSW: Specialist Palliative Care Social Worker; PCPT: Specialist Palliative Care Physiotherapist; PCA: Specialist Palliative Care Administrative Staff; PPC: Pediatric Palliative Care.

**Table 2:** Semi-structured question guide for professionals on the interdisciplinary pediatric palliative care team.

Research Areas	Questions
Care for children with palliative care needs and their families	How do you experience caring for children with incurable diseases whose prognosis is death before adulthood? How do you experience the relationship with the child and his/her family? Could you describe the relationship you establish with the children and families you care for? What do you think is the most relevant aspect of the relationship between PPC professionals and children and their families? What feelings do you have about caring for these children and their families? Do you consider that “living with death” in the course of your work has influenced you personally or professionally? How do you experience the worsening of children? How do you experience the death of children? Could you describe any experience that you consider important during the performance of your work, and explain how it may have affected you emotionally?
Self-perception of the professional role in PPC	How do you think families perceive your work within PPC? How do you consider that working at PPC has influenced you personally and professionally?
Communication and decision making	How is information handled and shared within the team? What is most relevant? How is the process of communication with the family? How is the decision-making process carried out?

PPC: Pediatric Palliative Care.

**Table 3:** Trustworthiness criteria.

Criteria	Techniques Performed and Application Procedures
Credibility	Investigator triangulation: team meetings were organized during the thematic analysis, the results were compared, and the final results were identified. Member checking: post interview participant member checking consisted of offering all participants the opportunity to review the audio or written records.
Transferability	In-depth descriptions of the study, providing data and describing the study design and its different sections (context, research team, reflexivity process, sampling, inclusion criteria, data collection, and analysis).
Dependability	Audit by an external researcher, responsible for the assessment of the study protocol, with a special focus on the method and process of implementation during the study.
Confirmability	Investigator triangulation, data collection triangulation. The process of reflexivity was conducted through the description of the researchers’ positioning; reflective debriefing by the researchers during data collection and analysis.

## Data Availability

The datasets generated and/or analyzed during the current study are not publicly available due to ethics restrictions but are available from the corresponding author on reasonable request.

## Supplementary Materials

The following supporting information can be downloaded at https://www.mdpi.com/article/10.3390/children10040700/s1: Table S1. Sociodemographic data of participants from the interdisciplinary PPC team; Table S2. Narratives from themes and sub-themes.
