# Supplementary material for: The Emotional Experience of Caring for Children in Pediatric Palliative Care: A Qualitative Study among a Home-Based Interdisciplinary Care Team
Source: Children (Basel). 2023 Apr 9;10(4):700. doi: 10.3390/children10040700 (PMC10136408; doi:10.3390/children10040700)
Supplement: Supplementary file 1 [file children-10-00700-s001.zip › children-2295698-supplementary.pdf]

The Emotional Experience of Caring for Children in Pediatric Palliative Care: A Qualitative Study among a Home-Based Interdisciplinary Care Team

Patricia Rico-Mena [1,2] Javier Güeita-Rodríguez [3,*], Ricardo Martino-Alba [4], Marina Castel-Sánchez [1] and Domingo Palacios-Ceña [3]

[1] Physical Therapy and Health Sciences Research Group, Faculty of Sport Sciences, Department of Physiotherapy, Chiropody and Dance, Universidad Europea de Madrid, 28670 Madrid, Spain
[2] International Doctorate School, Rey Juan Carlos University, 28008 Madrid, Spain
[3] Department of Physical Therapy, Occupational Therapy, Physical Medicine and Rehabilitation, Research Group of Humanities and Qualitative Research in Health Science, Rey Juan Carlos University, 28922 Alcorcón, Spain
[4] Pediatric Palliative Care Unit, Hospital Universitario Infantil Niño Jesús, 28009 Madrid, Spain
* Correspondence: javier.gueita@urjc.es

Table S1. Sociodemographic data of participants in the interdisciplinary PPC team.

**Participant code**	**Profession**	**Sex**	**Level of specific PC training**	**Time working at the PPC unit of NJH**	**Area or service where you previously worked**
PCD1	Pediatrician	Man	Masters	2 years	Pediatric intensive care and emergency care<br>Pediatric liver transplantation<br>Pneumology<br>Neonatology
PCD2	Pediatrician	Man	Masters	3 years	Neuropediatrics
PCD3	Pediatrician	Man	Masters	5 years	Pediatric Intensive Care
PCD4	Pediatrician	Man	Masters and PhD	10 years and 7 months	Pediatric intensive care, general pediatrics, pediatric trauma, pediatric oncology, pediatric oncology
PCD5	Pediatrician	Woman	Masters	5 years	Neuropediatrics
PCD6	Pediatrician	Woman	Masters	4 years	Pediatric emergency medicine and general pediatrics
PCD7	Pediatrician	Woman	Masters	6 years	Has always worked at PPC
PCN1	Nurse	Woman	Masters	6 years	Catalan Institute of palliative oncology for adults
PCN2	Nurse	Woman	Postgraduate course	7 years	Pediatric oncohematology service
PCN3	Nurse	Woman	Postgraduate course	11 years	Pediatric emergency care, neonatology, intensive care and pediatric oncology
PCN4	Nurse	Woman	Residency	15 months	Pediatric intensive care, general pediatrics, pediatric trauma, pediatric oncology, pediatric oncology
PCN5	Nurse	Man	Residency	3 months	Pediatric nursing

PCP1	Psychologist	Woman	Masters in family therapy/Basic course in PC	5 years	Oncohematology and pediatric hematopoietic transplant service and pediatric intensive care.
PCP2	Psychologist	Woman	Masters	10 years	Hematology and hemotherapy
PCSW1	Social worker	Man	Postgraduate course	4 years	Pediatric palliative care foundations and adult palliative care service
PCSW2	Social worker	Woman	Postgraduate course	10 years	Pediatric Oncology
PCPT1	Physical therapist	Woman	Postgraduate course	6 years	Respiratory physical therapy for healthy children and children with associated pathology
PCA1	Administrative staff	Woman	Basic course	11 years	Pathological anatomy, neurology and social pediatrics

PPC: Pediatric Palliative Care; PC: Palliative Care; NJH: Niño Jesús Hospital; PCD: Specialist Palliative Care Doctor; PCN: Specialist Palliative Care Nurse; PCP: Specialist Palliative Care Psychologist; PCSW: Specialist Palliative Care Social Worker; PCPT: Specialist Palliative Care Physiotherapist; PCA: Specialist Palliative Care Administrative.

Table S2. Narratives from themes and subthemes.

Theme 1. Changing life for the better.
Subtheme: Change of life perspective on life.
**Now I value more things in life**
EPE4: "On a personal level they have also given me a lot. Working in palliative care has really changed my life and has taught me to appreciate life, it has taught me to value what I have, because the children have shown me so much, so much happiness with what they have, the parents so much love for their children with all the problems they have, they have taught me to see life differently."<br><br>*EPE2: "It's like I'm grateful to feel a little bit like I have the opportunity. These people are going to pass away, I mean the child and his family, and they are going through something so difficult. And I am lucky enough, so to speak, to be the person who is going to be with them, to be able to attend to them and take care of them."*
**Personal and professional growth**
EPTS1: "…Working here has changed my way of being, my way of communicating, my way of respecting other cultures, other people with functional diversity, other acute situations, it has made me become very aware of what I see on a daily basis in order to respect society. Before, maybe I was more cocky, I used to pick on people more, being here working and being immersed in it all the time makes you mature in a way that would not happen to me in other places."<br><br>*EPM4: "It has made me learn about families, it has helped me to be more sensitive to suffering, it has also helped me to experience my own limitations, and that always adds up. This is a job that helps you develop personally."*

Subtheme: Compassion satisfaction.
**Feeling comforted**
EPPS1: "I am comforted by what we call satisfaction through compassion, in other words, it gives me more and I learn a lot about the meaning of life, which we are not afraid of on a daily basis, and with the children and families I say: "Wow, what bravery, what courage, what a struggle!"<br><br>*EPE2: "I don't know how to tell you, but the thing that most ties me here is the moment when they die and they die well. It makes a big impact on me when you are at the home and the child has passed away and the first thing they say to you is: thank you. That means that it went well because if we have a father who has lost his child and the first thing he does is to tell me -all my colleagues- that we have done well and that he is calm, for me that is the best thing and you are left with the feeling: we have worked hard, it has been difficult but it has turned out well."*
**Special bonds**
EPPS1: "We may be taking care of a child for ten years, and we have seen them teething, having their birthday and blowing out candles, going to school, and having brothers and sisters."<br><br>*EPM6: "One day we went to the zoo with 5 or 6 children and their families, we did a wheelchair race down one of the slopes and when we got to the bottom one of the girls choked with a phlegm from how much she had laughed, it was one of the most beautiful images I have. That and listening to music with some of the teenage girls from Oncology."*
**Theme 2: Adverse effects of work.**
Subtheme: Feeling scarred
**Enduring suffering**
EPM6: "Well, it has been very difficult, we have had a period with a lot of deaths that were grouped together. During my time in palliative care, every time a child has died, I am able to say whether he died at home or at the hospital, I can recall who was on duty, why he died, how he died, I have a very

photographic memory. At the time, I am unable to remember a lot of things and the feeling was that his little tribute to stay in my memory, could not be recorded because of the number of deaths that followed; that was hard for me. And among others, there was a child who was a donation in asystole, who came with a lot of dyspnea and had to wait to be intubated to be able to sedate him more, because if I sedated him and he stopped he would not be able to donate, that was perhaps one of the hardest situations."

EPM7: "For me the hardest thing is malpractice: when we ourselves, a colleague in our service is not giving adequate care which is detrimental to the child. Yes, I feel angry, and then there are also difficult moments, but even more rare is when you think that you're going to be able to control a symptom well and you can't control it."

Strategies to ease the emotional burden.

EPF1: "At the beginning when you start in the unit, I was very surprised by that: how they talk about death, they don't laugh about it, but they have to give it a touch of humor, it's impossible not to do so, because otherwise you can't live with that pain, all the years they have been working in the unit."

EPM4: "To work here you have to have a life outside, in other words, you have to have family, friends, hobbies, you can't look for what you don't have at home at work because what sustains you is always outside of here."

Subtheme: Experiencing bad deaths

Therapeutic obstinacy

EPTS2: "I used to work in Oncology, and I saw that children were dying very badly. Therefore, the suffering of seeing how badly the children were dying and how they were suffering, and how their families were suffering, motivated me to be here. They were dying in pain, without information, in the hospital, without their family members who were important to them…"

EPE3: "...I have had relationships with very special children and I have seen how they have died and how they have undergone a very hard process and ultimately have ended up very badly and in a very difficult situation, because I know that the oncologists and the nurses and all those in Oncology always try to be ready for whatever may come to help them through it, they never think that they are doing them harm, but eventually you realize that sometimes the therapies are too aggressive and it is a medical futility, not because of bad intentions, but sometimes we have to know how to stop and we do not know how to stop, and we go on and on until finally the child dies because he cannot go on and he dies with a blood line, with a lot of drugs, not being able to say goodbye to his parents because he is half comatose, or with a pain that sometimes cannot be controlled..."

**Living in a hospital**
EPM1: "Well, look, the hardest experiences I have had of children in palliative care were before I was in palliative care, when children who should have received palliative care were not receiving it because it did not even exist. And I do have painful experiences of children who have lived in intensive care for years. It still causes me a lot of pain to remember that because it was a terrible thing, for a child to live in an intensive care unit for three years until he dies, that is brutal." <br>*EPE4: "You used to see a patient come to a pediatric ward and ask you to help them because they had an appointment on Monday, another one the following month, and you say: "Oh, what a nuisance, they want me to fix their life for them. And you realize that these patients have a lot of overload, the parents have to go to many medical appointments."*
